# Internal Hernia in Pregnant Woman due to Congenital Transmesenteric Defect

**DOI:** 10.12669/pjms.37.5.4116

**Published:** 2021

**Authors:** Xu Yuansheng, Wang Yi, Fang Jinyan

**Affiliations:** 1 Xu Yuansheng, MD. Department of Emergency, Affiliated Hangzhou First People`s Hospital, Zhejiang University School of Medicine, Hangzhou 310006, China; 2 Wang Yi, MD. Department of Emergency, Affiliated Hangzhou First People`s Hospital, Zhejiang University School of Medicine, Hangzhou 310006, China; 3 Fang Jinyan, MD. Department of Emergency, Affiliated Hangzhou First People`s Hospital, Zhejiang University School of Medicine, Hangzhou 310006, China

**Keywords:** Internal Hernia, Pregnant Woman, congenital transmesenteric defect

## Abstract

Congenital transmesenteric hernias are uncommon and are a rare cause of bowel obstruction, which is even rarer in pregnant woman. Because of the lack of specific symptoms or reliable sensitive markers, it is difficult to diagnose internal hernia at early stage, therefore resulting in the delay of surgical intervention and a high mortality rate, especially in pregnant woman. We report a case in which a woman presenting at 16 weeks` gestation was admitted with symptoms of nausea, vomiting and left upper abdominal pain similar to her first-trimester morning sickness. Nephrolithiasis of the left kidney detected by ultrasound may lead to early incorrect diagnosis. Due to the patient`s concern about known adverse effects of ionizing radiation on the fetus, computed tomography was postponed until abdominal pain worsened, coffee color gastric contents vomited and anus stopped exhaust and defecation 12 hours later. Low dose CT plain scan showed features of small bowel obstruction by an internal hernia. Emergency exploratory laparotomy revealed a mesenteric defect of the left colon with a 30 cm long jejunal herniating distal to 10 cm of the ligament of Treitz. The involved small bowel was strangulated and gangrened, necrotic segmental resection and end to end anastomosis were performed subsequently, and the mesenteric defect was then successfully repaired with sutures.

## INTRODUCTION

The incidence of internal hernia is very low.^1^,^2^ Internal hernia accompanying pregnancy due to congenital transmesenteric defect is even more uncommon and is associated with a poor or complicated outcome, especially if early diagnosis is not established and surgical intervention is not undertaken. The main life-threatening complications comprise visceral obstruction, strangulation and gangrene of the herniated bowel, perforation and maternal or fetal death.^3^ A case of a pregnant patient with congenital transmesenteric defect leading to internal hernia is presented in this report.

## CASE REPORT

A 31-year-old woman with a 16-week gestation in her second pregnancy was admitted to our emergency department with a 2-hour history of nausea, vomiting, intermittent acute epigastric pain and left lumbago. She had normal temperature, normal anal exhaust and defecation on the morning of presentation, she also had no other comorbid conditions and had no any other surgical history but spontaneous abortion at 10^th^ week. She had no previous similar episodes of abdominal pain and medication. she also had no history of smoking and alcohol. During her first trimester, she had undergone severe morning sickness, alleviating gradually in her subsequent trimester.

**Fig.1:**
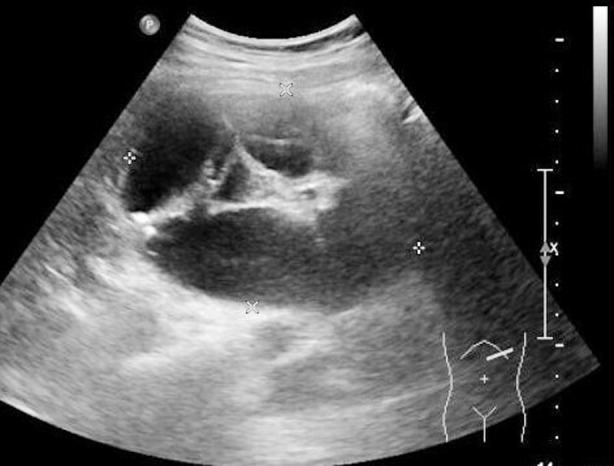
Ultrasonography shows dilated fluid-filled loops of bowel within a herniating mass.

**Fig.2:**
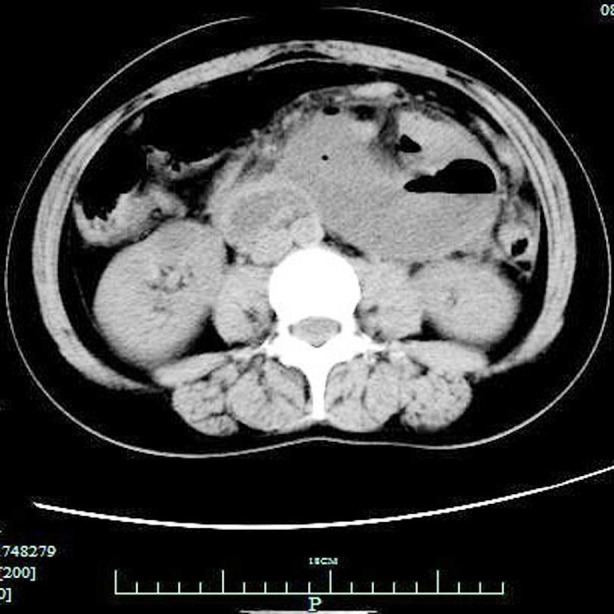
Axial CT image of abdomen demonstrates dilated small bowel loops in the left upper abdominal quadrant combined with air-fluid level.

**Fig.3:**
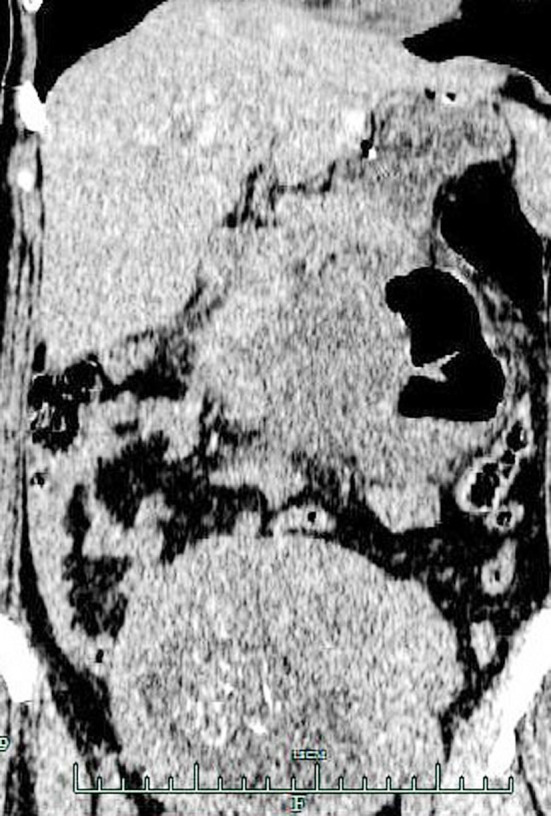
Coronal CT image of the abdomen shows dilated small bowel loops and air-fluid level with herniating mass. Gestational uterus is also seen.

**Fig.4:**
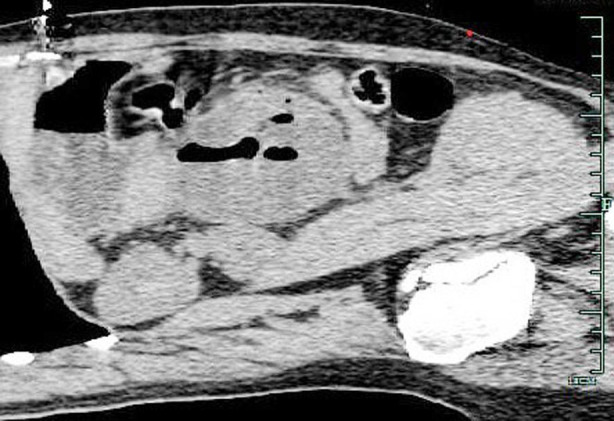
Sagittal CT image of the abdomen shows herniated small bowel with air-fluid levels.

Physical examination revealed normal vital signs. The abdominal exam showed soft abdomen with local tenderness in the left epigastrium and hypochondric ureteral regions. Rebound tenderness was negative, while left renal area percussion pain was positive. Bowel sounds were six beats/minute without gurgling. Laboratory findings were 11500/ml of white blood count (with 87.5% neutrophils), 7mg/L of highly sensitive C-reaction protein. The tests of liver function, kidney function, d-dimer, amylase, lipase, ionic level and triglyceride were all in the normal range. Lactate levels were slightly elevated to 2.4 mmol/L (reference range 0.4–2.0 mmol/L). The obstetrician on duty verified that the fetal heart rate and movements were normal. Electrocardiogram indicated sinus rhythm without ST-T segmental change. An abdominal ultrasound scan didn`t reveal any sign of biliary lithiasis, pancreatitis, appendicitis, seroperitoneum. The urinary ultrasound scan found multiple stones in her left kidney. Radiological examinations were refused. The renal calculus was considered to be the reason of epigastric pain and treated by intravenous phloroglucinol and magnesium sulphate, which once resulting in the relief of her abdominal pain. However, paroxysmal pain exacerbated gradually and became persistent pain. Then, coffee-like vomitus was throwing up and confirmed positive by occult blood test. The abdominal re-exam showed soft abdomen with diffuse and mild rebound tenderness in the left epigastrium. Bowel sounds were decreased. Point-of-care ultrasonography showed dilated fluid-filled loops of bowel (Fig 1). Then, low dose CT plain scan was permitted by the patient and her husband after 10 hours admission. The CT scan demonstrated dilated small bowel loops in the left upper abdominal quadrant combined with the small-bowel feces sign (Fig 2,3,4). A small amount of free intraperitoneal fluid was found surrounding the intestine. No free air was identified. There was no rupture or perforation in the visceral organs. The findings were suggestive of an internal herniation.

**Fig.5:**
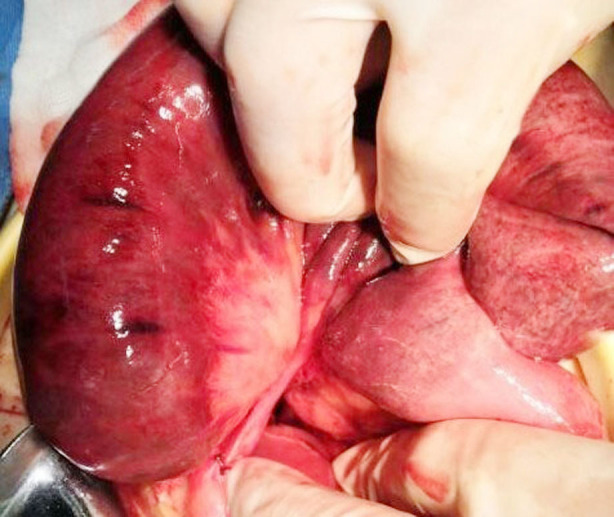
Intraoperative finding indicates a closed loop of the involved inviable small intestine.

The patient was transferred to the operating room, and exploratory laparotomy was performed immediately. It showed a large defect of left colonic mesentery, through which about 30cm of small bowel (beginning from 10 cm distal to the ligament of Treitz) had herniated and strangulated (Fig.5). The hernia was eliminated after the congenital defect was sutured, gangrenous bowel was resected, and end-to-end anastomosis was done. The postoperative course was uneventful. The patient recovered rapidly and was discharged home on postoperative day-7. The pregnancy has so far been proceeding normally.

## DISCUSSION

It is estimated that the incidence of internal hernia is 0.2-0.9%^2^, accounting for 0.6–5.8% of all cases of small bowel obstruction.^4^ Most internal hernias were caused by mesenteric defects due partly to surgical operations such as Roux-en-Y gastric bypass procedure.^3^ Congenital transmesenteric hernias are even less, constituting only 5–10% of internal hernias.^4^,^5^ The incidences of congenital transmesenteric defect and hernias in pregnant woman are unknown.

The acquired defect is usually attributed to abdominal surgery and traumatic laceration. In our case, the patient had no history of any trauma and surgery. Thus, mesenteric defect was regarded as congenital. The formation mechanism of congenital mesenteric defects was hypothesized including poor mesenteric degeneration of the dorsal mesentery, rapid expansion of mesenteric segment, and developmental enlargement of a hypovascular area.^5^

Pregnancy is associated with hernia formation. The enlargement of the uterus and increased abdominal pressure predispose patients to displacement of the small intestine and internal herniation as well as diaphragmatic hernia, incisional hernia.

The diagnosis of internal hernia is extremely difficult because there are no radiographic findings in early stage and no specific laboratory biomarkers to confirm the suspicion. Surgical exploration is the only means of definite diagnosis. A history of intermittent or chronic abdominal pain or distension associated with nausea and vomiting may be suggestive.^6^

The plain abdominal roentgenogram may show increased gas accumulation, air-fluid levels and stepladder arrangement. These signs of strangulation as a consequence of small bowel obstruction usually occur after several hours.^7^ These findings should warn the clinician of the possible diagnosis of an internal hernia and secondary intestinal obstruction.

Ultrasound has also been shown to be an effective tool for the diagnosis of bowel obstruction and hernias. According to a small-scale prospective study^8^, dilated bowel on ultrasonography is superior to x-ray with a sensitivity of 91% vs 46.2% and specificity of 84% vs 66.7% for diagnostic small bowel obstruction. Sonographic signs strongly suggestive of bowel obstruction include dilated fluid-filled loops of bowel (>25mm), to-and-fro movement of bowel contents, free fluid between the loops of bowel, edematous bowel wall (wall thickness >3mm), and loss of peristalsis.^9^ Reduced or absent color Doppler flow means strangulated intestinal necrosis requiring bowel resection.

CT examination is crucial in the diagnosis of patients suffering from internal hernia. Except for aforementioned nonspecific signs of bowel obstruction, there are some characteristic manifestations in contrast-enhanced CT including the swirl sign, the mushroom sign, the hurricane sign, the compressed superior mesenteric vein`s “breaking” sign and ectopic small bowel behind the superior mesenteric artery.^3^ However, the pregnant patient often excessively concerns about radiation exposure and the side effect of contrast associated with fetal harm, thus causing the rejection or delay of radiography. Actually, radiation exposure from a CT and X-ray is usually at a negligible dose far below 50 mGy safe for fetus, and intravenous iodinated contrast material is also generally judged to be safe during pregnancy.^10^ In present case, the fetal dose was far below this level. So radiographic tests should not be withheld from a pregnant patient when it is regarded as necessary and conducive to identify the underlying maternal pathologic condition.

MRI has the advantage of the lack of ionizing radiation. However, gadolinium contrast can pass through the blood-placental barrier and put the fetus under the potential risk.^3^ Moreover, MRI has no superiority over the CT scan in finding suggestive signs of internal hernia including the clustered loops, dilated and displaced bowel loops, or mesenteric edema.^3^

The differential diagnosis of an internal hernia is based on excluding other acute abdominal disease, including cholelithiasis, pancreatitis, urolithiasis, appendicitis, ankylenteron and so on, especially causes of small-bowel obstruction. In our case, the early diagnosis was confused by the absent previous surgery or trauma and the present findings of multiple stones with percussion pain over left kidney, and also retarded by the patient`s rejection of CT scan until aggravation of her condition. It’s regrettable that delayed diagnosis and exploration lead to small bowel necrosis. Fortunately, subsequent process was propitious.

Laparoscopy or exploratory laparotomy is needed in the patients with features of intestinal obstruction despite some cases of transient internal hernia can improve itself spontaneously. The potentially fatal complication consists of strangulated ileus, perforation, and death. The state of viability of the herniated bowel determines whether resection is necessary or not. The resection of the devitalized bowel is required if the involved bowel is nonviable. Furthermore, the elimination of the hernia, repair of the defect should be also included in surgical management. The prognosis depends on the development of further complications. Delayed diagnosis of internal herniation during pregnancy lead to raise the risk of maternal and fetal deaths.

## CONCLUSION

Internal hernia due to congenital transmesenteric defect is a rare cause of intestinal obstruction in pregnant woman. An internal hernia should be taken into account in a patient with bowel obstruction without previous abdominal surgery, adhesion or trauma. CT examination is crucial in the diagnosis of patients suffering from complications. Definite diagnosis relies on surgical exploration. Surgical management including resection of necrotic bowel and suture of the defect is indispensable, and prior to dwelling on diagnosis as the clinical features show surgical indications.

### Author Contributions:

**FJ:** Did manuscript writing.

**WY:** Edited the manuscript.

**XY:** Responsible and accountable for the accuracy or integrity of this study.

## References

[1] Ur Rehman Z, Khan S (2010). Large congenital mesenteric defect presenting in an adult. Saudi J Gastroenterol.

[2] Ghahremani GG (1984). Internal abdominal hernias. Surg Clin North Am.

[3] Warsza B, Richter B (2018). Internal Hernia in Pregnant Woman after Roux-en-Y Gastric Bypass Surgery. J Radiol Case Rep.

[4] Dowd MD, Barnett TM, Lelli J (1993). Case 02-1993:a three-year-old boy with acute-onset abdominal pain. Pediatr Emerg Care.

[5] Gyedu A, Damah M, Baidoo PK, Yorke J (2010). Congenital transmesenteric defect causing bowel strangulation in an adult. Hernia.

[6] Vaos G, Skondras C (2007). Treves'field congenital hernias in children:an unsuspected rare cause of acute small bowel obstruction. Pediatr Surg Int.

[7] Fujita A, Takaya J, Takada K (2003). Transmesenteric hernia:report of two patients with diagnostic emphasis on plain abdominal X-ray findings. Eur J Pediatr.

[8] Jang TB, Schindler D, Kaji AH (2011). Bedside ultrasonography for the detection of small bowel obstruction in the emergency department. Emerg Med J.

[9] Argintaru N, Al-Den A, Chenkin J (2015). Diagnosis of a Strangulated Laparoscopic Incisional Hernia with Point-of-Care Ultrasonography. West J Emerg Med.

[10] Tirada N, Dreizin D, Khati NJ, Akin EA, Zeman RK (2015). Imaging Pregnant and Lactating Patients. Radiographics.

